# Metabolic Scope and Interspecific Competition in Sculpins of Greenland Are Influenced by Increased Temperatures Due to Climate Change

**DOI:** 10.1371/journal.pone.0062859

**Published:** 2013-05-14

**Authors:** Henrik Seth, Albin Gräns, Erik Sandblom, Catharina Olsson, Kerstin Wiklander, Jörgen I. Johnsson, Michael Axelsson

**Affiliations:** 1 Department of Biological and Environmental Sciences, University of Gothenburg, Gothenburg, Sweden; 2 Department of Mathematical Sciences, University of Gothenburg, Gothenburg, Sweden; 3 Department of Neuroscience and Physiology, Sahlgrenska Academy, University of Gothenburg, Gothenburg, Sweden; Institute of Marine Research, Norway

## Abstract

Ongoing climate change has led to an increase in sea surface temperatures of 2–4°C on the west coast of Greenland. Since fish are ectothermic, metabolic rate increases with ambient temperature. This makes these animals particularly sensitive to changes in temperature; subsequently any change may influence their metabolic scope, i.e. the physiological capacity to undertake aerobically challenging activities. Any temperature increase may thus disrupt species-specific temperature adaptations, at both the molecular level as well as in behavior, and concomitant species differences in the temperature sensitivity may shift the competitive balance among coexisting species. We investigated the influence of temperature on metabolic scope and competitive ability in three species of marine sculpin that coexist in Greenland coastal waters. Since these species have different distribution ranges, we hypothesized that there should be a difference in their physiological response to temperature; hence we compared their metabolic scope at three temperatures (4, 9 and 14°C). Their competitive ability at the ambient temperature of 9°C was also tested in an attempt to link physiological capacity with behaviour. The Arctic staghorn sculpin, the species with the northernmost distribution range, had a lower metabolic scope in the higher temperature range compared to the other two species, which had similar metabolic scope at the three temperatures. The Arctic staghorn sculpin also had reduced competitive ability at 9°C and may thus already be negatively affected by the current ocean warming. Our results suggest that climate change can have effects on fish physiology and interspecific competition, which may alter the species composition of the Arctic fish fauna.

## Introduction

Over the last decade sea surface temperatures on the west coast of Greenland have increased by 2–4°C and the glaciers are decreasing in size at an unprecedented rate [1], [2]. This increase in temperature is believed to have severe physiological, behavioural and ecological implications for ectothermic animals, such as fishes, inhabiting these waters because metabolic rate [3], [4] and aerobic metabolic scope [5]–[8] may change with temperature. However, information on how elevated temperatures may disrupt species-specific temperature adaptations, e.g. behaviour or mechanisms at the molecular level, and concomitantly the competitive balance among coexisting species, due to changes in the capacity to undertake aerobically challenging activities [7], is scarce. The importance of studying species interactions to understand the consequences of climate change in the Arctic has recently been highlighted [9]. Metabolic rate is a key physiological factor linking physiology with behavior [10], and several studies have demonstrated that metabolic scope, the difference between resting and maximum metabolic rates, is reduced at an upper critical temperature in fishes [7], [11]. This is thought to constrain whole animal performance traits such as locomotion and digestion and lead to reduced fitness, but few studies have empirically demonstrated such linkages between aerobic performance and fitness.

**Figure 1:**
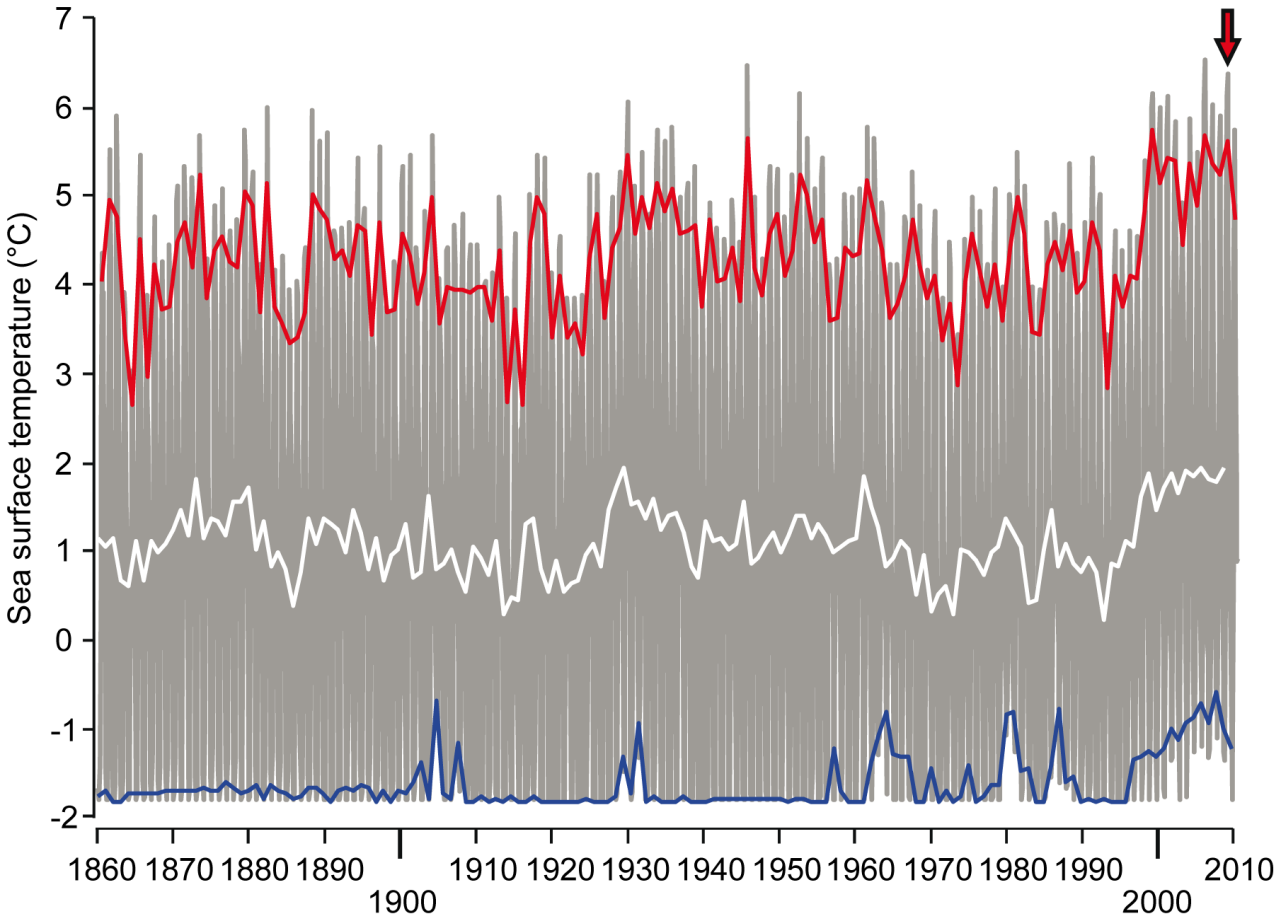
Sea surface temperatures outside Disko Island. Sea surface temperatures recorded by ships of opportunity between 1860 and the summer of 2010. The location was just outside of Disko Island at coordinates: Lat. 70N, Long. 54W. The white line represents the average temperature over the year and. the red and blue lines represent the average of the three warmest months (July, August, September) and the three coldest months (December, January, February), respectively. The red arrow denotes the summer temperature in August 2009 when the experiments were performed. Graph is based on *NOAA NCDC ERSST version2 - Improved extended reconstructed global sea surface temperature data based on COADS* (http://iridl.ldeo.columbia.edu/SOURCES/.NOAA/.NCDC/.ERSST/.version2/).

**Figure 2:**
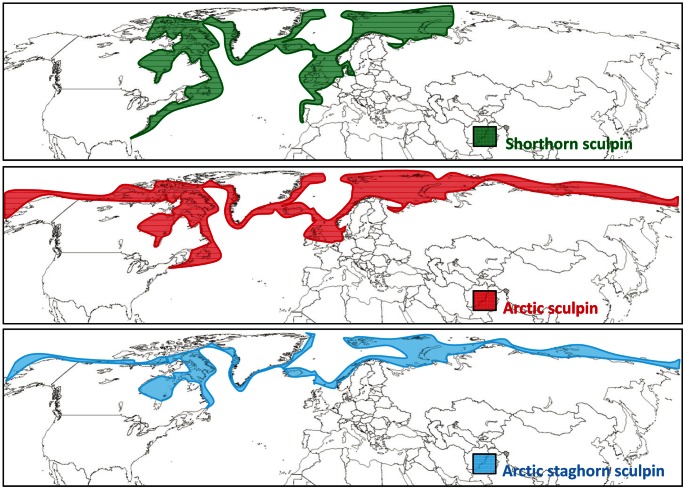
Geographical distribution of three species of sculpin. Shorthorn sculpin (*M. scorpius*) is distributed along the north Atlantic coastline as far south as Florida and Portugal. It also occurs in the Arctic north of Canada and Russia. Arctic sculpin (*M. scorpioides*) has a more northerly distribution and is mainly found throughout the Arctic along the northern shores of Canada and Russia, although low numbers are occasionally found south of Newfoundland and Scotland. The Arctic staghorn sculpin **(** *G. tricuspis* **)** has an even narrower distribution and is entirely restricted to Arctic waters. Distribution data are accessed through GBIF Data Portal, data.gbif.org and www.iobis.org.

This study was performed in August 2009 at the Danish Arctic station at Qeqertarsuaq, in the Disko Bay area on the west coast of Greenland. This area is affected profoundly by on-going climate change and sea surface temperature (SST) records proximate to Disko Bay reveal a clear deviation from historic patterns after the mid-1990s, when temperatures started to increase [2]. The SST in August 2009 was the third highest recorded since temperature monitoring began 150 years ago (Fig. 1) and stable for the duration of the study and any variation depended more on the depth and location than on a daily variation and the lowest temperature recorded was 6°C at the bottom off shore. The maximal temperature is reached in August and is elevated throughout the summer months. In the present study, we assessed the effects of temperature on metabolic scope and the ability of three species of sculpins, the Arctic staghorn sculpin (*Gymnocanthus tricuspis*), Arctic sculpin (*Myoxocephalus scorpioides*) and shorthorn sculpin (*Myoxocephalus scorpius*), inhabiting the coastal waters of Disko Bay to compete for a common resource, in the form of a cover (i.e. shelter):. The geographical distribution of these species differs considerably, particularly in latitudinal range; with the Arctic staghorn sculpin having the narrowest and northernmost distribution, the Arctic sculpin having an intermediate distribution range and the shorthorn sculpin having by far the widest latitudinal distribution (Fig. 2).

Given the strictly Arctic distribution of the Arctic staghorn sculpin, we hypothesized that this species would be most sensitive to increases in temperature. We therefore predicted that this species would be less tolerant and thus unable to maintain aerobic scope after an increase in temperature. All three species are sit-and-wait predators and comparatively poor swimmers that coexist in the littoral zone in shallow waters and on rocky bottoms, where they are preyed upon by a variety of fish and bird species. Furthermore, since these three species are of similar size, their relative ability to compete for access to similar types of cover should be of considerable ecological relevance [12]. We assessed the ability to compete for helter since marine sculpins are territorial and use a cover when available [13], hypothesizing that the species with the largest metabolic scope would have a competitive advantage, as metabolic capacity provides the physiological basis for physical stamina required in competition.

## Materials and Methods

### Animals

Shorthorn sculpin (*Myoxocephalus scorpius*) (N_tot_ = 29; 30–735 g), Arctic staghorn sculpin (*Gymnocanthus tricuspis*) (N_tot = _19; 79–187 g) and Arctic sculpin (*Myoxocephalus scorpioides*) (N_tot_ = 19; 30–215 g) were caught off the coast of Qeqertarsuaq (Disko Island) on the west coast of Greenland. All three species were caught in similar habitats, i.e. shallow rocky bottoms. Fish were held at 9°C for at least one week before experimentation in a recirculating water system, where ca. one third of the water volume was replaced on a daily basis to ensure good water quality. The holding temperature was selected to mimic the ambient water temperature at the time of the experiments.

### Ethics Statement

This study was carried out in strict accordance with the recommendations of the Swedish Board of Agriculture and conformed to the national guidelines of Sweden. All experiments were approved by Inuussutissarsiornermut Suliffeqarnermullu Naalakkersuisoqarfik, Departementet for Erhverv og Arbejdsmarked Uumasut nakorsaqarnerisigut immikkoortortaqarfik Veterinærafdelingen (Danish Veterinary Unit of Greenland), ethical permit *Dyreforsøg/1-09*. The experiments, as well as fish handling, were designed to minimise disturbance to the animals. The animals were caught by rod and line and after the experiments all animals were returned to their natural environment. No samples (blood/tissue) were taken from the animals. The land accessed was not privately owned or otherwise protected. The study did not involve any endangered or protected species.

### Respirometry

Oxygen consumption (MO_2_) was measured in resting fish and after forced exercise at three temperatures (4, 9 and 14°C) using intermittent closed respirometry. The respirometers (7.9 L each) were submerged in an outer 240 L tank supplied with aerated seawater. A submersible pump ensured continuous mixing of the water inside the respirometer. Another pump controlled by a time relay was used to close the respirometer for 15–20 min during MO_2_ measurements. The relative change in oxygen content (%) was measured with an oxygen meter (Oxi 340i, WTW, Weilheim, Germany) placed in-line with the mixing pump. After a habituation period of 24 h during which we continually monitored oxygen consumption, resting and maximal MO_2_ was measured at 9° then at 4°C and 14°C. Resting MO_2_ was measure 1 h after reaching each temperature and maximal MO_2_ was achieved by manually chasing the fish inside the respirometers for 10 min. Temperature was changed at a rate of ca. 1°C h^−1^, so there was a recovery period of at least 5 h between each test. To control for confounding effects of diurnal variations or repeated treatments, MO_2_ was measured again at 9°C at the end of each trial. The respirometers were regularly cleaned using hot water to remove any microbial growth and background oxygen consumption was subtracted by measuring MO_2_ at the different temperatures after each experiment.

MO_2_ was calculated using the following formula:
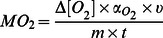
where Δ[*O_2_*] is the relative difference in oxygen content (%) after closing the respirometer for 15–20 min; α_O2_ is the oxygen content of water at the particular temperature, salinity and barometric pressure (*on average* 4°C: 11.08 mg l−^1^; 9°C: 9.84 mg l^−1^; 14°C: 8.84 mg l^−1^); *v* is the volume of the closed circulation excluding the fish, *m* is body mass and *t* is time. Due to differences in size between species, MO_2_ was normalized to the body mass for a 100 g fish [3], [5], [14]:



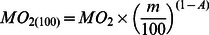
where *MO_2(100)_* represents the oxygen consumption of a 100 g fish and *MO_2_* is the oxygen consumption of a fish with a body mass *m*, as calculated above. *A* is the mass exponent that describes the scaling of metabolic rate with mass. Theoretically the value of *A* varies between 0.6 and 0.9 and we used a mass exponent of 0.8 [3], [5], [14].

### Behavioural Experiments

Competitive ability was assessed in fish staged in dyads to compete for access to protective cover. Two size-matched individuals (difference in body mass <10%) of different species were placed in a 80 L tank, containing aerated seawater at a temperature of 9°C, and allowed 24 h to recover from any handling stress. A protective cover, i.e. a hollow plastic tube sized to fit one sculpin only, was placed in the tank. Fish were allowed 60 min to investigate the the hollow plastic tube before a standardized predator attack was simulated by a plastic heron beak, whisking through the experimental area for 10 s, which should increase the motivation of the fish to seek and maintain cover [12]. The subsequent interaction between the two competitors was recorded for 30 min using a digital camcorder (DCR-SR72E, Sony Corp. Tokyo, Japan) mounted above the experimental tank. The success of the two individuals in each tank was then ranked according to a protocol where the individual spending most time in the cover was rewarded one point and an individual that managed to force the other competitor out, and consequently take over the cover, was rewarded two points. The scoring was based on the assumption that a takeover is a more challenging act, revealing the true competitive capacity of the animal. We did not observe any overt aggression (e.g. biting), nor any threat displays. Instead, competition for cover appeared to occur by attempting to push the cover holder out, alternatively trying to avoid being pushed out, using physical stamina.

### Statistics

**Figure 3:**
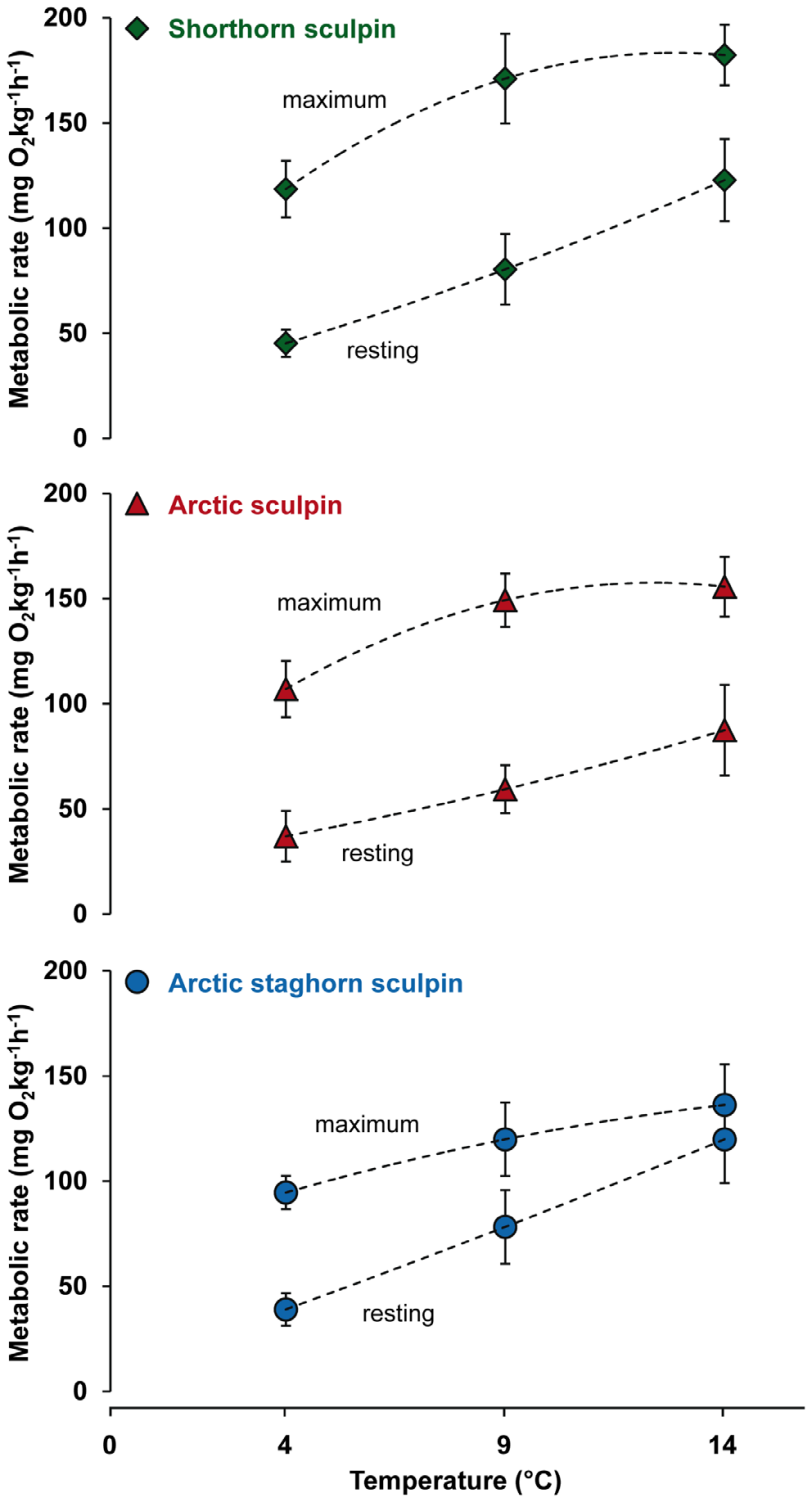
Resting and maximal metabolic rates in three species of sculpin. Oxygen consumption rates in shorthorn sculpin (*M. scorpius*; green, n = 9), Arctic sculpin (*M. scorpioides*; red, n = 9) and Arctic staghorn sculpin **(** *G. tricuspis*; blue, n = 9**)** acutely exposed to 4, 9 and 14°C. The acclimation temperature and prevailing sea water temperature when the experiments were conducted in 2009, was 9°C. The data represent values from the statistical model and the error bars represent the 95% confidence interval.

**Figure 4:**
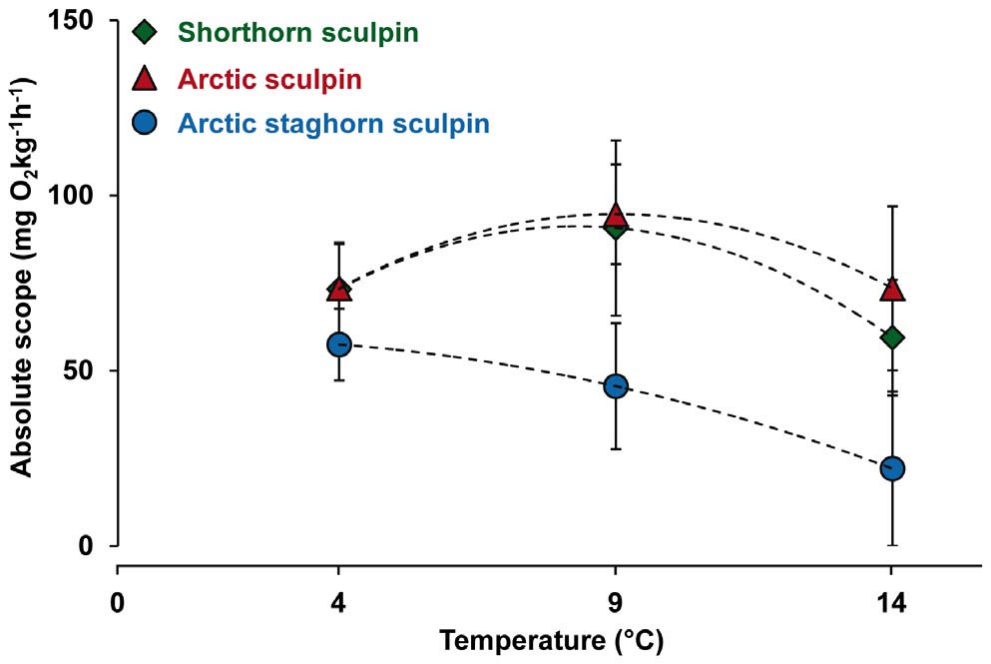
Metabolic scope in three species of sculpin. Metabolic scope calculated as the difference between the resting and the maximal oxygen consumption rates in Shorthorn sculpin (*M. scorpius*; green, n = 9), Arctic sculpin (*M. scorpioides*; red, n = 9) and Arctic staghorn sculpin **(** *G. tricuspis*; blue, n = 9**).** Fish were acutely exposed to 4, 9 and 14°C. The acclimation temperature and prevailing sea water temperature when the experiments were conducted in 2009, was 9°C. The data represent values from the statistical model and the error bars represent the 95% confidence interval.

The metabolic scope experiments were designed such that each fish was exposed to all temperatures, resulting in repeated measurements and dependent observations. We used the compound symmetry model, meaning that observations taken from the same fish have a constant covariance, independent of the time aspect. The explanatory variables *species* and *temperature*, as well as their interaction, were used in the model. Non-significant interactions were excluded from the final model. A check of the residuals revealed no deviations from the assumptions connected to the statistical methods. A significant difference was assumed when p<0.05. The p-values were corrected for multiple testing. For MO_2_, the 95% confidence intervals are illustrated with error bars in Figs. 3–4. The calculations were done using Statistical Analysis Software (SAS Institute Inc. Cary, North Carolina, USA). In the behavioural experiments, a Sign test was used to reveal significant differences in the success of gaining and retaining cover.

## Results and Discussion

Both resting and maximal metabolic rate increased with temperature (4 to 14°C), with the increase in resting metabolic rate being more pronounced for all three species, i.e. 171% (max: 54%), 135% (max: 45%) and 205% (max: 43%) for the shorthorn, Arctic and Arctic staghorn sculpins, respectively (Fig. 3). In shorthorn and Arctic sculpins this meant that the aerobic scope leveled off as temperature increased, resulting in bell-shaped scope curves for these two species. This is similar to a previous hypothesis that states that there is an optimal temperature^7^, below and above which there are limitations in e.g. growth and reproduction. Nevertheless, maximal metabolic scopes for shorthorn and Arctic scuplins were reached at 9°C (91±13 and 95±7 mg O_2_ kg^−1 ^h^−1^, respectively) (Fig. 4), which was the recorded SST at the time of the experiment. For Arctic staghorn sculpin, however, metabolic scope decreased from its peak at 4°C (57±5 mg O_2_ kg^−1 ^h^−1^) and was much reduced at 14°C (22±11 mg O_2_ kg^−1 ^h^−1^), probably limiting the capacity for metabolically demanding activities beyond basic maintenance. Overall, Arctic staghorn sculpin had a significantly lower metabolic scope at all temperatures compared to the other two species (Fig. 4). Thus, this species may already have exceeded its optimal temperature during the warm summer of 2009. The available temperature data (Fig. 1) shows that there has been a rapid increase in temperature over the last years, while the distribution (Fig. 2) of this species has been restricted to the arctic region. Therefore, although the exact temperature range of the Arctic staghorn sculpin is unknown, it is reasonable to speculate that its optimal temperature is at or below 4°C.

Some ectotherms can maintain a low resting metabolism by actively avoiding high temperatures and thereby avoid negative influences on metabolic scope, e.g. as observed in Atlantic cod (*Gadus morhua*) when swimming in a thermally stratified water column [15]. However, this may not be an option for the benthic, stationary sculpins studied here. An alternative strategy is to reduce the temperature dependence of metabolism *per se,* maintaining metabolic rate despite an increase in temperature, i.e. acclimation. This is a common strategy for fish to cope with environmental challenges such as hypoxia [16] and is also seen in the gadiod *Lota lota* in response to high summer temperatures [17]. Our preliminary, unpublished data support the existence of a metabolic depression in association with warm-acclimation in sculpins. In gadiods, such acclimation depends on rapid down-regulation of enzymes involved in aerobic metabolism [17]. Although the suppression of metabolic rate could be a long-term process, none of the sculpins maintained the resting metabolism seen at 4°C when exposed to 9°C, despite the latter being the prevailing temperature when they were caught. Hence, the sculpins would have had time to lower the metabolism to maintain a lower resting metabolism at 9°C. Given the plasticity of thermal acclimation, the resting metabolism seen at 4°C could have increased if the fish were allowed more time to acclimate to this temperature (as during winter). Such cold adaptation, although a matter of debate, was demonstrated by Precht et al. [18] and could depend on mechanisms at the whole animal level, as well as the enzyme level [19]. The physiological responses to acute temperature increases are probably of high ecological importance in these fish and may be particularly important in their coastal habitats, e.g. as a result of diurnal and/or tidal cycles [20]. In fact, several studies indicate that short-term temperature fluctuations and heat spells will increase in magnitude and frequency as a consequence of global warming [21], and studies on e.g. amphibians [22] indicate that it is not the increase in average temperature *per se*, but rather the greater short-term temperature variability that will be most detrimental for many populations. However, few studies in fish have addressed this question [23], [24].

**Figure 5:**
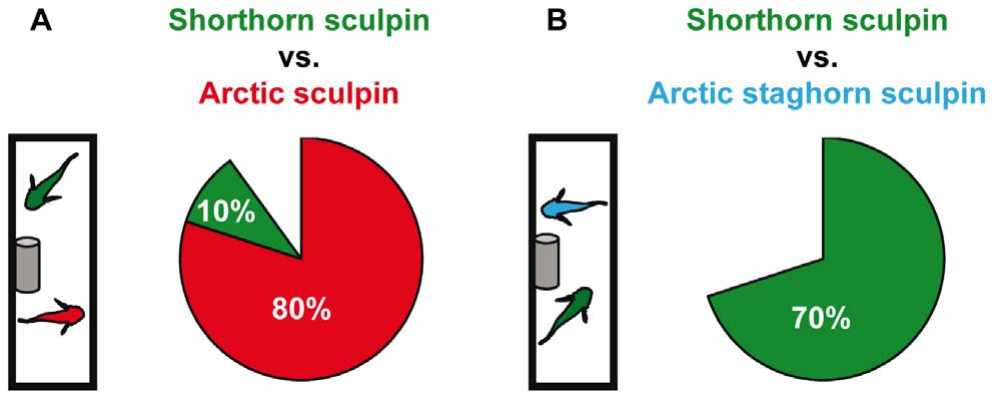
Competitive ability. Illustration of how competitive ability of individual fish was assessed. A predator attack was simulated to create a situation where the fish would have to compete in order to gain access to a common recourse (i.e. the cover). Shorthorn sculpin (*M. scorpius*, green, n = 20), Arctic sculpin (*M. scorpioides* red, n = 10) and Arctic staghorn sculpin **(** *G. tricuspis*, blue, n = 10**)** were placed in size-matched pairs in an 80 L tank containing aerated seawater at 9°C. A shorthorn sculpin was allowed to compete against either an Arctic sculpin or an Arctic staghorn sculpin. Ten pairs were tested for each species combination.

The results of the behavioral aspect of the study were partly consistent with our second hypothesis that metabolic scope can be used to predict competitive ability. Arctic staghorn sculpin, the species with the lowest metabolic scope at 9°C, was consistently outcompeted by shorthorn sculpin for access to cover (Fig. 5), whereas Arctic sculpin outcompeted the shorthorn sculpin despite a similar metabolic scope (Fig. 3–4). The fact that Arctic staghorn sculpin, also had the lowest competitive ability of the three species supports the metabolic theory suggested by Brown [10], stating that metabolism, and metabolic rate in particular, shape population dynamics and predator/prey interactions.

The common resource was a protective cover since marine sculpins use a cover (i.e. shelter) when available [13] and the performance of the sculpins was monitored after a simulated attack, which should increase the motivation of the fish to regain and maintain a cover [12]. Numerous species of fish increase their use of a cover/shelter by up to 40% when the risk of predation is high [12], [25], and in a pilot study it was apparent that a simulated attack increased cover/shelter use in all three species. The cover was not added to the arena until just before the simulated attack. This was done in order to exclude a “resident effect” whereby the prior resident may win due to higher motivation to fight, i.e. the value asymmetry hypothesis [26]. It should be pointed out that we could not control for potential species differences in the innate motivation to seek cover. A higher motivation could potentially enhance the competitive ability when the physiological capacity (metabolic scope) is equal. For example, the less developed body armour of Arctic sculpins could make them more vulnerable to some (e.g. smaller sized) predators compared to the shorthorn sculpin, thereby increasing the motivation to seek cover [27]. This might explain why Arctic sculpin outcompeted shorthorn sculpin in competition for cover, despite a similar metabolic scope at 9°C.

Previous studies on shorthorn sculpin have shown that the ability to both capture prey and to escape predators is affected by acclimation temperature [28], [29]. These performances were improved during acclimation to higher temperatures (15°C), while cold acclimation (5°C) had the opposite effect. While these studies did not report on metabolic scope, it is possible that the sculpin were able to maintain or even elevate their metabolic capacity when acclimated to the higher temperature, suggesting considerable potential for phenotypic plasticity in terms of metabolic capacity when exposed to more long-term thermal challenges. Interestingly, the escape behavior of another related sculpin species, the long-spined sea scorpion (*Taurulus bubalis*), which has narrower geographical distribution and does not occur as far south as the shorthorn sculpin, was not improved by acclimation to higher temperatures [29]. Instead, acclimation to low temperatures was advantageous for this species when performing in cold water. This indicates that temperature acclimation patterns may differ even between closely related species. Such differences may be of major importance for interactions among species coexisting in habitats with large seasonal fluctuations in temperature, a fact that needs to be considered when interpreting results from only one acclimation temperature.

Climate change is a driving force for evolution and the influence of temperature on metabolism is an important mechanism linking physiology and behavior with fitness in ectothermic animals [30], [31]. Learning more about thermal tolerance and its physiological, behavioral and ecological consequences will aid in understanding the possible effects of climate change. While the future of many species will depend on their capacity to find new habitats and/or to adapt to changes within their present habitats [32], [33], the capacity to do so may be limited in species like the Arctic staghorn sculpin that is already pushed to the higher end of its physiological thermal optimum as indicated in this study. Thus, in due course there will likely be a shift in the species distribution, abundance and migratory patterns as our oceans warm [34].
